# Does the Food Ingredient Pectin Provide a Risk for Patients Allergic to Non-Specific Lipid-Transfer Proteins?

**DOI:** 10.3390/foods11010013

**Published:** 2021-12-21

**Authors:** Hanna Steigerwald, Frank Blanco-Perez, Melanie Albrecht, Caroline Bender, Andrea Wangorsch, Hans-Ulrich Endreß, Mirko Bunzel, Cristobalina Mayorga, Maria José Torres, Stephan Scheurer, Stefan Vieths

**Affiliations:** 1 Paul-Ehrlich-Institut (PEI), Federal Institute for Vaccines and Biomedicines, 63225 Langen, Germany; hanna.steigerwald@pei.de (H.S.); frank.blanco@pei.de (F.B.-P.); melanie.albrecht@pei.de (M.A.); andrea.wangorsch@pei.de (A.W.); stefan.vieths@pei.de (S.V.); 2 Karlsruhe Institute of Technology (KIT), Institute of Applied Biosciences, 76131 Karlsruhe, Germany; caroline.bender@kit.edu (C.B.); mirko.bunzel@kit.edu (M.B.); 3 Herbstreith & Fox KG, Neuenbürg, Turnstrasse 37, 75305 Neuenburg, Germany; h.u.endress@herbstreith-fox.de; 4 Allergy Unit, IBIMA-Regional University Hospital of Malaga-UMA, 29010 Malaga, Spain; mayorga.lina@gmail.com (C.M.); mjtorresj@gmail.com (M.J.T.)

**Keywords:** food allergy, pectin, non-specific lipid transfer protein, nsLTP, Pru p 3

## Abstract

Pectin, a dietary fiber, is a polysaccharide that is widely used in food industry as a gelling agent. In addition, prebiotic and beneficial immunomodulatory effects of pectin have been demonstrated, leading to increased importance as food supplement. However, as cases of anaphylactic reactions after consumption of pectin-supplemented foods have been reported, the present study aims to evaluate the allergy risk of pectin. This is of particular importance since most of the pectin used in the food industry is extracted from citrus or apple pomace. Both contain several allergens such as non-specific lipid transfer proteins (nsLTPs), known to induce severe allergic reactions, which could impair the use of pectins in nsLTP allergic patients. Therefore, the present study for the first time was performed to analyze residual nsLTP content in two commercial pectins using different detection methods. Results showed the analytical sensitivity was diminished by the pectin structure. Finally, spiking of pectin with allergenic peach nsLTP Pru p 3 led to the conclusion that the potential residual allergen content in both pectins is below the threshold to induce anaphylactic reactions in nsLTP-allergic patients. This data suggests that consumption of the investigated commercial pectin products provides no risk for inducing severe reactions in nsLTP-allergic patients.

## 1. Introduction

Pectins are heterogeneous, highly complex polysaccharides, which accumulate in the middle lamella and in primary cell walls of higher plants, with larger amounts being found in primary cell walls of dicotyledonous plants as compared to monocotyledonous plants. In plants it plays an important role as hydrating agent and cementing material [1,2]. In general, pectins consist of a backbone composed of homogalacturonan (HG), rhamnogalacturonan-I (RG-I) as well as rhamnogalacuronan-II (RG-II) and in some cases xylogalacturonan (XGA) regions [3]. HG regions consist of α-(1-4)-linked-d-galacturonic acid (GalA) units that can be acetylated at their hydroxyl groups in position C-2 and/or C-3 and methyl-esterified at their carboxyl groups present at C-6 [4]. The ratio of methyl-esterified GalA groups to total GalA groups is quantified as degree of esterification (DE) [5]. Pectin can be classified as high methoxyl pectin (HMP) with a DE > 50% or as low methoxyl pectin (LMP) with DE < 50% [6].

Based on their physicochemical characteristics, pectins are increasingly used in the field of food technology as emulsifiers, stabilizers, and thickening agents as well as in cosmetics and in medicinal products [7]. As a water-soluble fiber, it is used in various food products such as jam, yoghurt drinks, fruity milk drinks, and ice cream [8,9]. Due to potential health benefits, pectins are also added as nutritional supplements [10]. Several studies reported beneficial effects of a high-fiber diet on aspects such as physical bowel function [11,12], diabetic control, and cholesterol levels [13,14] as well as inflammation [15,16] and carcinogenesis [17]. Recent studies also examined the effects of pectin on allergic sensitization by modulating the gut microbiota [18,19], which showed beneficial effects of pectin supplementation on allergies [20].

However, some cases of anaphylactic reactions after consumption of pectin supplemented foods were reported [21,22,23]. Anaphylactic reaction after ingestion of a pectin-containing yogurt pouch occurred in a patient with food allergies to peanut, tree nuts, and shellfish [23]. Additionally, development of anaphylaxis after consumption of a pectin-containing smoothie was reported in a patient with known allergies to cashew nut and possibly pistachio as well as hypersensitivity to grapefruit [22]. However, the allergy-eliciting agent and the underlying mechanism of these allergic reactions have not yet been identified. Pectins are frequently produced from fruits containing allergenic non-specific lipid-transfer proteins (nsLTPs), e.g., nsLTP Mal d 3 in apples and Cit l 3 in lemons [24]. These small, heat-stable, structurally highly conserved proteins belong to the major fruit allergens [25]. nsLTPs are the most frequent cause of food allergies in adults in the Mediterranean area [26,27] and are known as panallergens, as patients often present IgE binding to several different nsLTPs due to cross-reactivity, leading to a phenomenon also known as the LTP-syndrome [28].

It was shown that sensitization to nsLTPs is particularly important in food allergy to Rosaceae fruits, although nsLTP-sensitized patients are commonly allergic to a wide variety of plant foods also including non-Rosaceae fruits, tree nuts, and vegetables [29]. The symptoms of allergic reactions can vary from oral allergy syndrome (OAS) via urticaria and angioedema to life-threatening anaphylactic reactions [30]. The peach nsLTP Pru p 3 was identified to be the primary sensitizer in most patients sensitized to nsLTPs and is therefore proposed as a marker allergen for nsLTP sensitization [31]. Due to the high cross-reactivity among the members of the nsLTP family, the use of Pru p 3 would provide insights into the possible reactivity to other members of the allergen family.

Two health claims have been granted for pectins in the EU: (I) Reduction of the blood glucose rise after meals and (II) maintenance of normal blood cholesterol levels after consumption of at least 6–10 g pectin per meal [32]. To achieve the high consumption of pectin required for these positive health effects as well as for potentially beneficial effects on allergies, consumption of foods enriched with pectin or of nutritional supplements containing pectins is required. To ensure safety of nsLTP-allergic patients in this context, information on the levels of allergenic nsLTPs in commercial food grade pectins is needed. Therefore, the present study aimed to detect potential nsLTPs in the pectin preparations and thereby assess the risk of pectin to induce severe reactions in nsLTP-allergic patients [33].

## 2. Materials and Methods

### 2.1. Material

**Figure 1:**
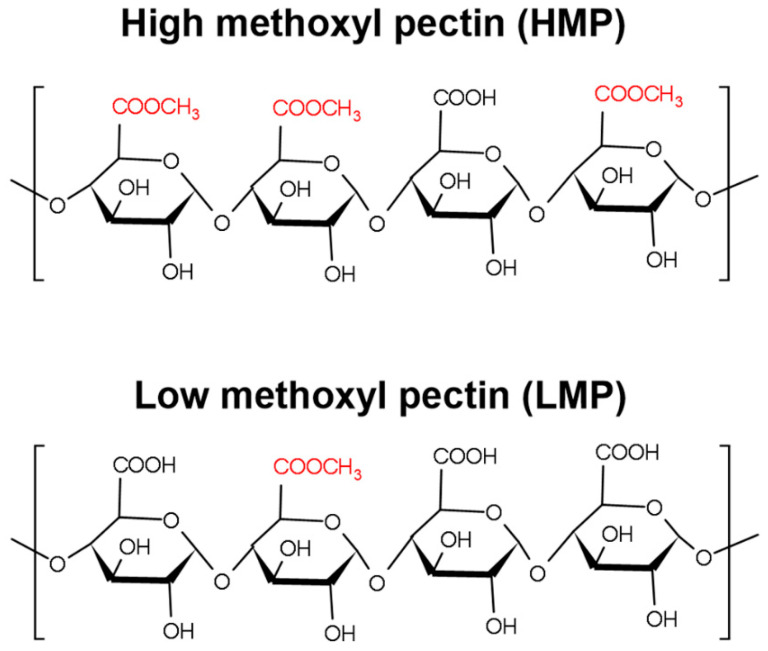
Scheme of the chemical structure of the heteropolysaccharide pectin indicating differences in the degree of esterification (DE). For chemical structure KingDraw 2.1 was used.

Pectins were provided by Herbstreith & Fox KG, Neuenbürg, Germany. As apple-derived pectin (AP), Herbapekt SF 50-LV a HMP with a DE of 57% and a low molecular weight (MW) was used. The used citrus-derived pectin (CP) was Classic CU901, a LMP with DE of 7.3% and a low MW (max 15 kDa) (Figure 1).

To catalyze pectin degradation Frutase^®^ PL (EC. 4.2.4.10) and Fruktozym^®^ P6-XL (EC. 3.2.1.15) were provided by Erbslöh Geisenheim GmbH (Geisenheim, Germany). Natural Pru p 3 (nPru p 3) was purified from freshly prepared peach peel as described previously [34]. Briefly, fresh ripe peaches were obtained from a local grocery store. The peel (250 g) was homogenized with a food processor (Bosch, Munich, Germany) in 400 mL of extraction buffer (2% PVPP, 2 mM EDTA, 10 mM DIECA in 10 mM potassium phosphate buffer) according to the method of Björkstén et al. [35]. To precipitate a substantial amount of naturally occurring pectin, CaCl_2_ was added to a final concentration of 80 mM and the mixture was incubated overnight at 4 °C. After centrifugation and subsequent filtration steps from 5 µm to 0.45 µm, the clarified extract was dialyzed three times overnight against 50 mM NaOAc, pH 5.3. nPru p 3 was subsequently purified from the filtrate by two step chromatography. First the extract was applied to a HiPrep™ SP HP 16/10 cation exchange column (Cytiva, Munich, Germany) using 50 mM NaOAc pH 5.3 as running buffer with 0 to 1 M NaCl gradient for elution. nPru p 3 containing fractions were further purified by size exclusion chromatography (HiPrep™ 26/60 Sephacryl S-100 HR column, Cytiva) and PBS (137 mM NaCl, 2.7 mM KCl, 10 mM Na_2_HPO_4_ and 1.8 mM KH_2_PO_4_; pH 7.4) as running buffer. Fractions containing the pure protein were pooled and total protein content was determined using the BCA assay (Fisher Scientific, Schwerte, Germany). Purity of nPru p 3 was assessed by Coomassie-stained (GelCode Blue, Fisher Scientific) SDS-PAGE and intact secondary structure by means of circular dichroism.

### 2.2. Methods

#### 2.2.1. Pectin Sample Preparation

To prepare 5% or 10% pectin solutions, the respective amount of pectin (*w*/*v*) was solubilized in either H_2_O at RT for 4 h under continuous stirring or in Fruktozym^®^ or Frutase^®^ solution (1:30 in H_2_O (*v*/*v*)) for 0.5, 4 or 16 h at 40 °C under continuous stirring, as indicated in the respective figure legend.

#### 2.2.2. Estimation of the Analytical nsLTP Threshold

Evidence for nsLTP concentrations leading to anaphylaxis or OAS were empirically obtained from the results of a clinical study (unpublished data). The thresholds for nsLTP consumption per meal are assumed as 300 µg for possible induction of anaphylactic reactions and 10 µg for induction of OAS.

#### 2.2.3. Pru p 3 Specific IgE ELISA

For Pru p 3 specific IgE (sIgE) ELISA, 96-well plates were coated with decreasing concentrations of nPru p 3 (10–0.05 µg/mL), nPru p 3 (10–0.05 µg/mL) spiked in 5% pectin or unspiked 5% pectin dissolved in 50 mmol/L sodium carbonate buffer, pH 9.6 overnight at 4 °C. After blocking with 10% fetal calf serum (FCS) in PBS, murine anti-Pru p 3 polyclonal serum (mice immunized with nPru p 3) was added (diluted 1:10) and incubated at 4 °C overnight. A biotin anti-mouse IgE antibody (Clone R35-118; BD Biosciences, Heidelberg, Germany) was incubated at RT for 1 h, followed by 30 min incubation with horseradish peroxidase-labeled streptavidin (BD Biosciences, Heidelberg, Germany). After addition of the substrate (0.525 mM 3,3′,5,5′-tetramethylbenzidine, 0.01% H_2_O_2_ in 0.21 M potassium citrate buffer; pH 3.95), the absorbance was measured at 450 nm. Graph Pad Prism version 8.4.2 was used for analysis of the area under the curve (AUC).

#### 2.2.4. SDS-PAGE and Immunoblotting

Pectin and protein samples were separated by SDS-PAGE under non-reducing conditions as described elsewhere using a 16% acrylamide gel [36] and visualized using GelCode Blue Stain Reagent (Fisher Scientific, Darmstadt, Germany).

For immunoblotting, nPru p 3 and/or pectin samples were subjected to SDS-PAGE under non-reducing conditions and transferred to a nitrocellulose membrane by semidry blotting [37]. After blocking (TBS buffer containing 0.3% Tween 20) the membrane was incubated with Pru av 3-reactive (cherry nsLTP) rabbit serum (CE-Immundioagnostika, Eschelbronn, Germany). Non-reactive rabbit pre-immune serum was used as control. To detect bound IgG, an HRP-labelled goat anti-rabbit IgG antibody (Cat. #7074, Cell Signaling Technology, Leiden, The Netherlands) and enhanced chemiluminescence (ECL) (Merck KGaA, Darmstadt, Germany) were used for visualization. AUC was analyzed using ImageJ version 1.53c, and values were normalized using blank values. Blank values were set as 0, the positive control nPru p 3 was set as 1.

#### 2.2.5. Size Exclusion Chromatography

For size exclusion chromatographic analysis (SEC, Merck Hitachi L-700), enzymatically degraded pectin samples were centrifuged, and supernatants were injected. A combination of TSKgel G2500PWxl and TSKgel G3000PWxl columns (both 300 × 7.8 mm, 7 µm particle size, Tosoh Bioscience GmbH, Germany) with an appropriate guard column, sodium nitrate (50 mM) as eluent (0.35 mL/min, 35 °C), and refractive index detection (RI, L-7490 LaChrom RI, Merck, Germany) were used. Molecular weight “calibration” was performed by using dextrans as standard compounds.

#### 2.2.6. Protein Precipitation

Protein precipitation from the pectin matrix was performed as reported by Niu et al., with slight modifications [38]. Briefly, ice-cold acetone was added to a 10% enzyme-treated pectin solution (ratio 4:1 (*v*/*v*)) and incubated at −20 °C overnight. Samples were centrifuged for 10 min at 15,000× *g*, and supernatants were discarded. Protein pellets (upper layer) were re-suspended in PBS without disturbing the pectin pellets (bottom layer) and immediately used in the posterior assays.

#### 2.2.7. Pectin Precipitation

Pectin precipitation was done using CaCl_2_ as reported by Lević et al. [39]. Briefly, 10% Fruktozym^®^-treated pectin was precipitated by adding CaCl_2_ to a final concentration of 100 mM and incubated overnight at 4 °C under continuous shaking. The samples were then centrifuged at 15,000× *g* for 10 min, and supernatant containing the protein were collected and immediately used in the posterior assays.

#### 2.2.8. β-Hexosaminidase Release from Humanized Rat Basophil Leukemia (huRBL) Cells

The mediator release assay was performed following the protocol established by Vogel et al. [40]. Briefly, huRBL-30/25 cells were harvested at the stationary phase, seeded 1 × 10^5^ cells/well in a 96-well plate and incubated overnight with human Pru p 3 IgE-reactive serum (specific IgE = 21.5 kU_A_/l referring to CAP class 4). After washing, cells were stimulated with antigen (nPru p 3, nPru p 3 spiked in 5% pectin or 5% pectin). Initially, 1.5 µg/mL nPru p 3 or 0.05 µg/mL nPru p 3 were used, representing the suggested anaphylactic and OAS thresholds for 5% pectin solution, followed by serial dilutions. The specific mediator release triggered by cross-linking of receptor-bound IgE on the surface of the huRBL cells was measured as extinction at 405 nm and estimated as percent of total β-hexosaminidase release obtained by lysing the cells with Triton X-100. The application of human serum in the study was approved by the local ethical committee of the Institut Universitari Dexeus of Barcelona (Spain) and informed consent was obtained from the patients. The sera were taken from a former study [41].

#### 2.2.9. Statistical Analysis

The results are represented as means ± standard deviation, and the data was statistically evaluated by ANOVA with Dunnett’s multiple comparison test (α = 0.05). The statistical software was Graph Pad Prism version 8.4.2.

## 3. Results

### 3.1. Detection of nsLTP Is Hampered in the Pectin Matrix

**Figure 2:**
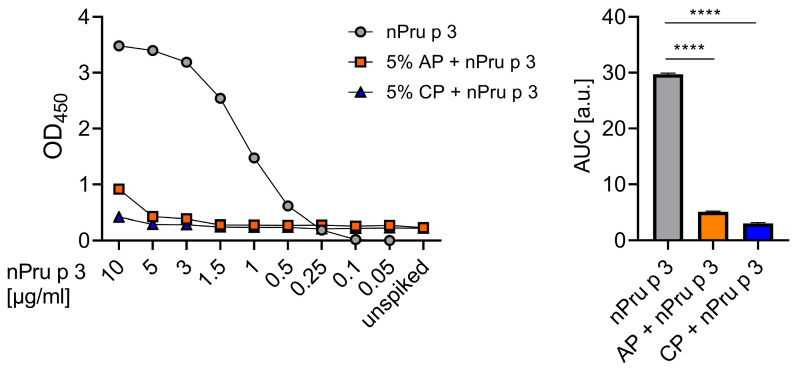
Detectability of nsLTPs in 5% apple-derived pectin (AP) and 5% citrus-derived pectin (CP) was evaluated by Pru p 3 (peach nsLTP)-sIgE ELISA. The nsLTP detectability was compared using the area under the curve (AUC). **** = *p* < 0.0001.

To analyze potential residual nsLTP content, two commercial pectins with low MW but different DE and source materials were used in this study (AP and CP). First, the nsLTP protein concentrations detectable in a pectin-based matrix were evaluated. Therefore, decreasing concentrations of nPru p 3, nPru p 3 spiked in 5% pectin, and unspiked 5% pectin were analyzed by Pru p 3-sIgE ELISA (Figure 2). Twice the value of the buffer control (resulting in OD_450nm_ of 0.08) was defined as limit of detection for positive results, allowing detection of allergenic protein down to 0.25 µg/mL. Using nPru p 3, a dose dependent standard curve and a maximum OD_450nm_ of 3.5 with the highest concentration (10 µg/mL) was obtained. In comparison, the maximum OD_450nm_ reached with nPru p 3 spiked into AP was around 1 and around 0.4 with nPru p 3 spiked in CP. In line with this, the AUC of both spiked pectins showed a significant decrease compared to nPru p 3 (Figure 2).

**Figure 3:**
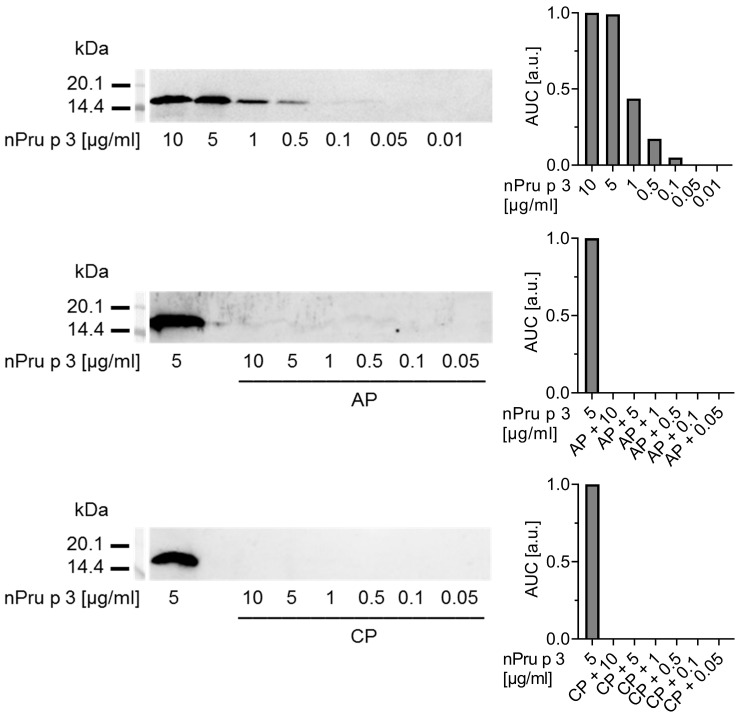
Detectability of peach nsLTP Pru p 3 in 10% pectin (apple-derived pectin (AP) and citrus-derived pectin (CP)) matrix was evaluated via immunoblotting using a cross-reactive nsLTP rabbit antiserum. The protein detectability was semi-quantified using the area under the curve (AUC) normalized to blank.

Similar results were obtained in immunoblots with nsLTP specific IgG using a rabbit antiserum (Figure 3).

Decreasing concentrations of nPru p 3 (10–0.01 µg/mL) and nPru p 3 dose-dependently spiked in 10% pectin were analyzed. Again, twice the value of the blank control was defined as positive signal. With this method, 0.1 µg/mL nPru p 3 were detectable as the lowest level from a standard curve without pectin matrix, whereas when spiked into AP or CP, the sensitivity was dramatically decreased as none of the added nPru p 3 concentrations could be recovered. The optical density of each band was semi-quantitatively analyzed as AUC.

### 3.2. Enzymatic Pre-Treatment of Pectin Increases the Analytical Sensitivity for nsLTP Detection

**Figure 4:**
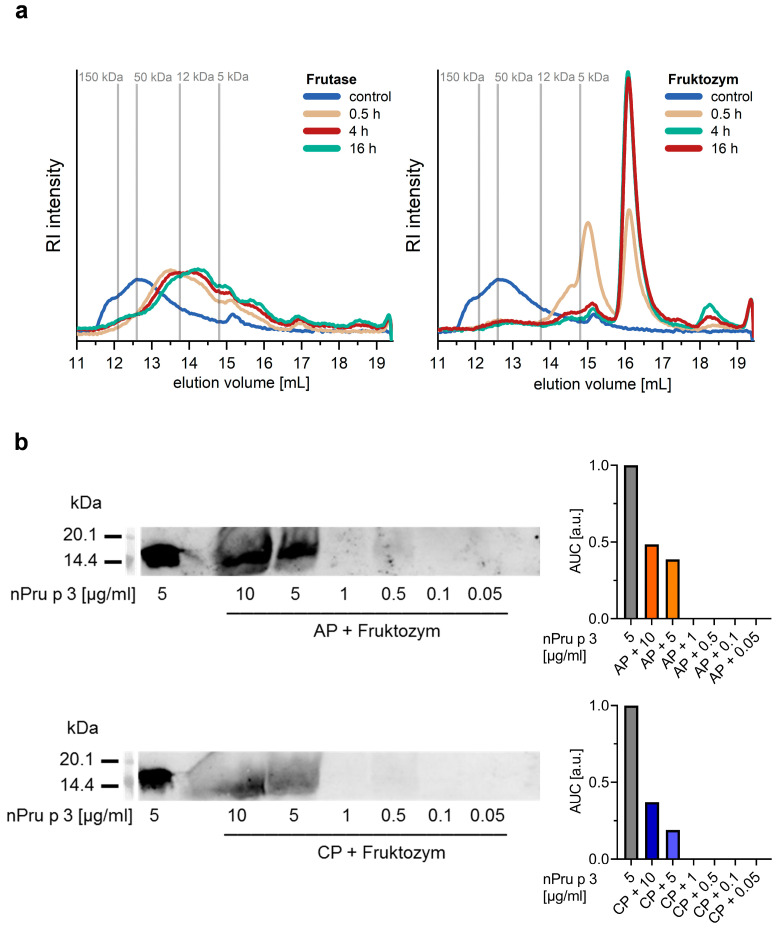
Size exclusion chromatography (SEC) coupled with refractive index (RI) detection derived chromatograms of AP treated with Frutase^®^ (**left**) and Fruktozym^®^ (**right**) over different incubation times and an untreated control. Dextrans with different molecular weights were used as markers (vertical lines) (**a**). Detectability of nPru p 3 in Fruktozym^®^-treated 10% apple-derived pectin (AP) and 10% citrus-derived pectin (CP) was analyzed via immunoblotting using a cross-reactive nsLTP rabbit antiserum. nPru p 3 (5 µg/mL) without pectin was used as control. The protein detectability was semi-quantified using the area under the curve (AUC) normalized to blank (**b**).

In an attempt to overcome the interference of the nsLTP detection by pectins, samples were subjected to enzymatic digestion. Therefore, two pectolytic enzymes, Fruktozym^®^ and Frutase^®^ were analyzed for their digestion efficacy of AP (Figure 4a).

SEC data indicated that incubation of AP with Fruktozym^®^ for a minimum of 4 h led to almost complete digestion of the polymeric pectin structures, resulting in oligomers with MW below 5 kDa (as estimated by using dextrans as molecular weight markers) as indicated by the increasing SEC peak with an elution volume of 16–17 mL (Figure 4a, right). In comparison, incubation with Frutase^®^ resulted only in low levels of oligomers with an apparent MW between 20 and 5 kDa that appeared after just 0.5 h. However, further cleavage over time was not detected (Figure 4a, left). Both enzymes increased the solubility of AP and CP as compared to the solubility in water (Figure S1a). A maximum of up to 20% AP was soluble within a time period of ca. 100 min using Frutase^®^ and within ca. 60 min using Fruktozym^®^ as compared to a maximum of 10% AP soluble after ca. 130 min in H_2_O. Both enzymes enabled dissolution of up to 20% CP in ca. 5 min as compared to a maximum solubility of 10% CP after around 70 min in H_2_O. Detectability and IgG-reactivity of nPru p 3 were neither affected by the enzymatic treatment nor by incubation at 40 °C for 6 h (Figure S1b).

Pectin degradation using Fruktozym^®^ prior to immunoblotting increased the analytical sensitivity of the method (Figure 4b). Decreasing concentrations of nPru p 3 were spiked in 10% AP and CP and subsequently treated with Fruktozym^®^. After the enzymatic treatment, a concentration of 5 µg/mL spiked nPru p 3 was detectable in both pectins via immunoblot with a recovery of about 40% in AP and 20% in CP calculated semi-quantitatively by AUC.

### 3.3. Enzymatic Treatment of AP Allows Detection of nsLTP Corresponding to Clinical Thresholds

To assess the possible risk for elicitation of OAS and anaphylaxis for nsLTP-sensitized patients, thresholds of allergen leading to the aforementioned clinical reactions needed to be estimated for the pectin samples. The calculation was performed in consideration of a clinical study addressed to evaluate positive effects of pectin consumption in food allergies. As the peach nsLTP-allergic patients will consume a smoothie containing 10 g pectin, the potential risk of allergic reactions needed to be clarified. To mimic the conditions of the clinical study, the amount of nsLTP in pectin samples leading to a risk for severe reactions for allergic patients was estimated as follows: an ingest of 10 g pectin in 100 mL smoothie (100 mg/mL) refers to a total pectin consumption by the patients in the planned clinical study. To avoid anaphylactic reactions, the amount of nsLTP in 10 g pectin should not exceed 300 µg nsLTP (3 µg per ml of the 10% pectin smoothie) (unpublished data). Accordingly, the threshold for induction of OAS was estimated to be 0.1 µg per ml of 10% pectin smoothie.

**Figure 5:**
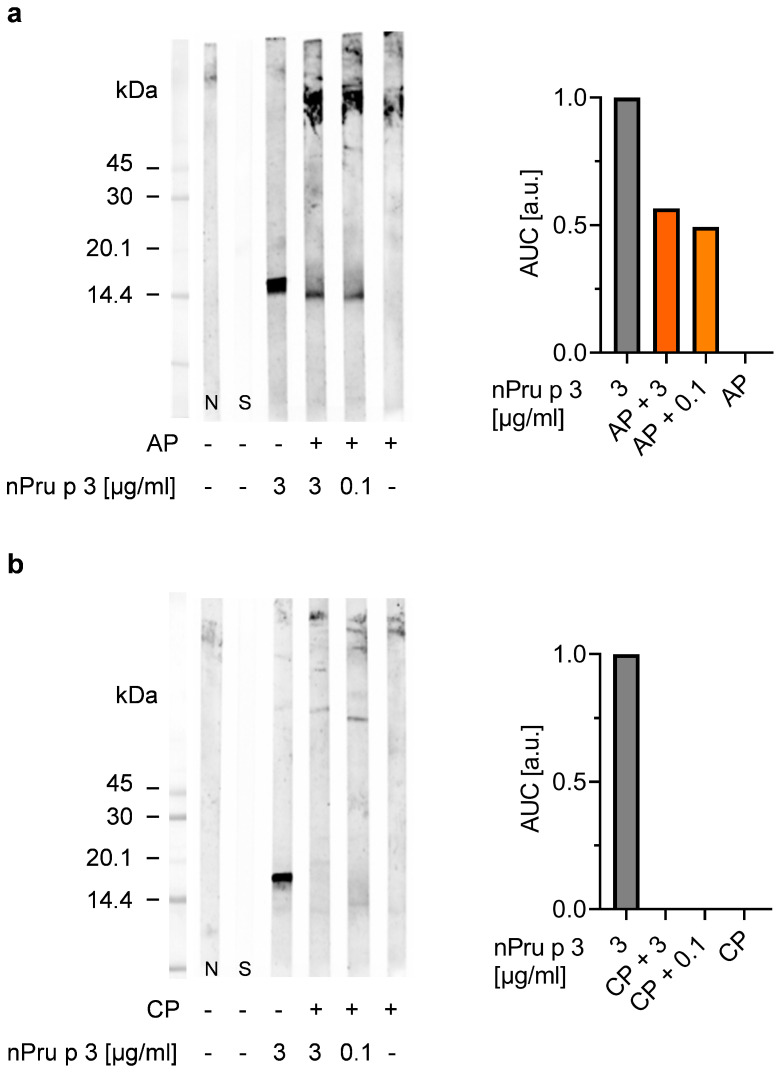
Detection of nPru p 3 spiked in 10% Fruktozym-treated apple-derived pectin (AP) (**a**), or citrus-derived pectin (CP) (**b**) via immunoblotting. The spiking concentrations represent the nsLTP thresholds eliciting anaphylactic (3 µg/mL) or oral symptoms (0.1 µg/mL). Detected bands were analyzed semi-quantitatively as area under the curve (AUC) normalized to blank.

In the following, detectability of nPru p 3 representing the suggested nsLTP thresholds eliciting oral (0.1 µg/mL) or anaphylactic reactions (3 µg/mL) spiked in 10% Fruktozym^®^-treated AP or CP was examined via immunoblot and was compared with unspiked pectin samples. Purified nPru p 3 (3 µg/mL) served as positive control (Figure 5). The results showed that in AP, both spiked nPru p 3 concentrations were detectable whereas no protein band was visible in the unspiked pectin (Figure 5a). We conclude the potential residual nsLTP content in AP is less than 0.1 µg/mL (or max 10 µg in 100 mL smoothie) and therefore does not likely provide a relevant risk for individuals with an nsLTP-allergy. Nevertheless, an interference of pectin is still recognizable, as the AUC of 3 µg/mL nPru p 3 spiked in AP is almost 50% decreased compared to the control. When spiked in CP none of the nPru p 3 was recovered (Figure 5b). Consequently, a risk assessment for CP will not be feasible.

### 3.4. Precipitation of Protein and Pectin Allows Detection of nsLTP Concentration in Pectin Corresponding to Threshold for Anaphylactic Reactions

**Figure 6:**
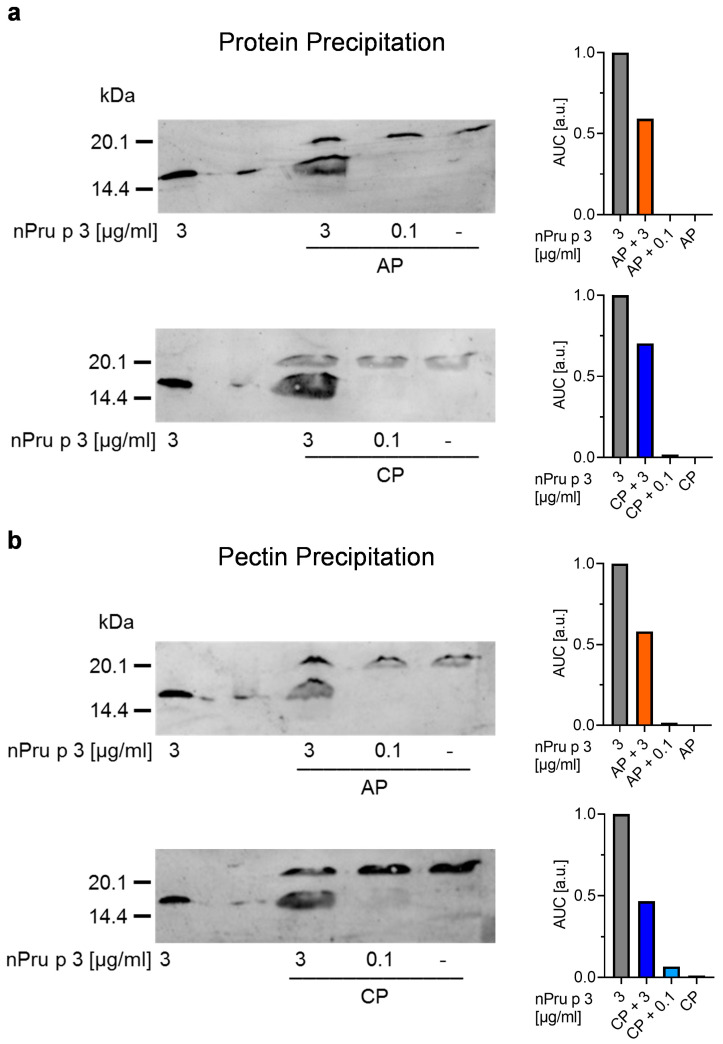
Precipitation of protein (**a**) or pectin (**b**) and subsequent analysis via immunoblotting. nPru p 3 concentrations representing the anaphylactic (3 µg/mL) or oral allergy syndrome (OAS) threshold (0.1 µg/mL) spiked in 10% Fruktozym-treated pectins, unspiked pectins and purified nPru p 3 were used for precipitation. The protein detectability was semi-quantified using the area under the curve (AUC) normalized to blank.

As a further approach to improve the detection of proteins in the Fruktozym^®^-treated pectin samples, protein precipitation using acetone or pectin precipitation using CaCl_2_ were performed (Figure 6).

The precipitations were done with Fruktozym^®^-treated 10% AP and CP spiked with 3 µg/mL (suggested threshold for anaphylactic reactions) or 0.1 µg/mL (suggested threshold for OAS) of nPru p 3, purified nPru p 3, as well as unspiked pectin. Subsequently, the samples were analyzed using immunoblot. AUC was used for semi-quantitative analysis. The results of protein precipitation and subsequent analysis of the precipitated protein showed that the spiked nPru p 3 concentration representing the anaphylactic threshold of 3 µg/mL was detectable in both pectins (Figure 6a), whereas the concentration representing the OAS threshold was not detectable. After precipitation of pectin with CaCl_2_ and subsequent analysis of the supernatant via immunoblot, the spiked nPru p 3 concentration representing the anaphylactic threshold was detectable in both pectins. At most, a very weak protein band was seen in CP spiked with nPru p 3 representing the OAS threshold of 0.1 µg/mL. In both unspiked pectin samples no protein band was visible (Figure 6b).

### 3.5. huRBL Assay Allows Detection of Spiked nPru p 3 Concentrations

**Figure 7:**
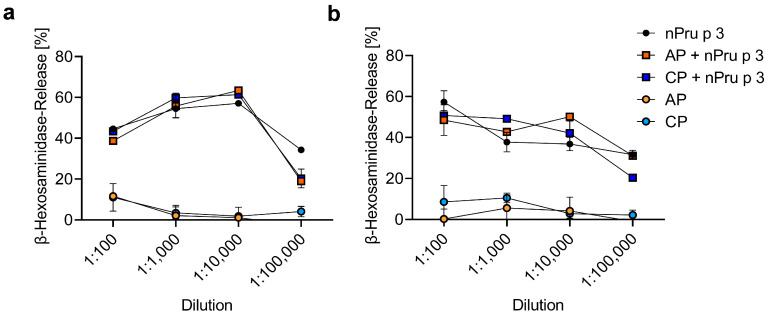
Mediator release assay using huRBL-30/25 cells passively sensitized with nsLTP specific IgE. Stimulation was done using enzyme-treated 5% apple-derived pectin (AP) and citrus-derived pectin (CP), either unspiked or previously spiked with nPru p 3 representing the anaphylactic (**a**) or oral allergy syndrome (OAS) (**b**) threshold added to the assay in dilution series.

To further increase the sensitivity of protein detection in a pectin-based matrix, a functional cellular assay (huRBL cells) was used to determine possible residual nsLTP. Thus, nsLTP concentrations eliciting anaphylactic or oral symptoms of nPru p 3 spiked in AP (5%) or CP (5%), nPru p 3 without pectin or pectin alone, all Fruktozym^®^-treated, were used for stimulation (Figure 7).

To exclude unspecific mediator release due to the enzymatic treatment, the spontaneous β-hexosaminidase release after incubation with Fruktozym^®^-treated pectin or Fruktozym^®^ alone was examined using a ten-fold dilution series from undiluted to 1:1000 (Figure S2a). The results showed that enzyme-treated pectin as well as enzyme alone induced almost 90% mediator release when added undiluted. This unspecific mediator release was reduced to a minimum (less than 5%) using higher dilutions of 1:100 or 1:1000. Furthermore, using a dilution series with highest concentration of 1:100, the enzymatic treatment did not show an interference with the detection of nPru p 3 effects (Figure S2b). nPru p 3 concentrations representing the suggested thresholds (1.5 µg/mL and 0.05 µg/mL for 5% pectin) were added either without or after pre-incubation with Fruktozym^®^ to the assay (Figure S2b). The results showed no important differences in mediator release between the enzyme-treated and the untreated nPru p 3 in both concentrations and all dilutions used (Figure S2b). The results of the huRBL assay after incubation with nPru p 3 and/or pectin samples indicated that the spiked nPru p 3 concentration representing anaphylactic threshold (Figure 7a) or OAS threshold (Figure 7b) were fully recovered. There was no substantial difference in the dose-dependent performance of the mediator release of nPru p 3 spiked in pectin compared to nPru p 3 without pectin. Unspiked pectin samples did not cause mediator release at the concentrations tested in the dilution series leading to the conclusion that its nsLTP content was below the threshold for clinical reactions.

## 4. Discussion

Pectin plays an important role as a food ingredient [8], which is not only due to its characteristics as thickening agent and stabilizer but also because of its reported health benefits after consumption of a certain amount [1,10,11,19]. Accordingly, pectin has attracted interest in the field of allergy prevention [19]. In the present study, the potential risk of pectin for nsLTP-allergic patients due to potential accumulation of residual allergens during the commercial production of pectins from sources such as apple or citrus fruits was evaluated. In many patients sensitized to nsLTPs, consumption of even minimal amounts of the allergen can cause symptoms such as diarrhea, urticarial, or even life-threatening anaphylactic reactions [30]. The average daily pectin intake from a regular or even high fiber diet was stated to be 2–38 mg/kg bodyweight resulting in consumption of 2.6 g pectin assuming an average bodyweight of 70 kg and a high pectin diet [42]. Thus, the allergen risk assessment of pectin is particularly important with regard to patients consuming higher amounts of pectin (5–10 g pectin per meal), e.g., as a nutritional supplement, as it is proposed to achieve the stated health claims [32].

In this study, we intended to establish methods for the detection of residual nsLTPs in a commercial apple-derived and a citrus-derived pectin preparation to test for their presence at levels able to cause clinical reactions after consumption of up to 10 g of pectin. The threshold that may trigger reactions was derived from dose-dependent food challenge experiments that will be published elsewhere as part of a clinical trial.

In agreement with the findings of the present study, it has been reported that the pectin-based matrix interferes with allergenic proteins. In in vitro experiments pectin was shown to protect kiwi allergens from digestion [43]. These results prompted us to apply two enzymes for pectin degradation to enhance the release of allergens and, thus, reduce the matrix effect. Incubation of AP with Fruktozym^®^, a mixture of several different enzymes, showed a decrease of the molecular weight of the apple-derived pectin from 50–150 kDa to below 5 kDa. In contrast, Frutase^®^, a pectin lyase catalyzes cleavage of α-(1,4) glycosidic linkages between methyl-esterified GalA units, and the activity therefore depends on the DE and on the distribution of the methyl esters [44]. Thus, the efficiency of the enzymatic digestion might also differ between HMP and LMP. In comparison, a mixture of different enzymes as present in Fruktozym^®^ is not limited to one cleavage site and might therefore have an increased efficiency.

After Fruktozym^®^-treatment, both spiked nPru p 3 concentrations were detectable in AP by immunoblot even though the pectin-based matrix still interfered with the method. In a CP-based matrix, none of the spiked nPru p 3 concentrations was detectable. These differences in protein detectability between the two pectins might occur due to structural differences, in particular the DE, as both pectins have a low MW. It has been reported that the DE of pectin affects the formation of pectin-protein complexes suggesting that the interaction of the protein is higher with a pectin of low DE [45]. Furthermore, differences in the gelling characteristics might lead to differences in the matrix effects hampering the protein detectability [46].

Following the hypothesis that the pectin matrix covers and masks the protein added to model samples, precipitation of either protein or pectin out of the solution could overcome this limitation. As the results showed, precipitation of protein as well as precipitation of pectin enabled the detection of the spiked nPru p 3 concentration representing the threshold for anaphylactic reactions in both pectins. This supports the assumption that the pectin matrix affected detectability of the allergenic protein.

Functional analysis of spiked nPru p 3 in pectin via the huRBL assay led to mediator release comparable to the nPru p 3 control without pectin. Due to their functional similarity to mast cells, RBL-30/25 cells sensitized with allergen-specific IgE are widely used for the determination of biological activity of allergens [47]. This indicates that even if the pectin-based matrix might hamper the detectability of residual nsLTPs, e.g., in immunoblotting and ELISA, their allergenic potential remains.

Remarkably, in the huRBL assay no interference of the pectin matrix was observed. The data of this study suggests that the detectability of proteins as well as the sensitivity of the methods used differ due to the structure of the pectins studied, in particular with respect to the DE. In any case, the spiked nPru p 3 concentration representing the threshold suggested for anaphylactic reactions was detected in both pectin preparations using different methods. However, and most importantly, in none of the methods used was residual nsLTP detected in the commercial pectin samples, suggesting that the amount of putative nsLTPs content in these specific commercial pectin preparations is below the threshold that can trigger anaphylactic reactions.

In summary, the results suggest that pectins studied from both apples and citrus do not pose a risk of causing allergic reactions in fruit allergic individuals sensitized to nsLTP, even after consumption of 10 g pectin in a single serving. Furthermore, at least 16 nsLTP allergic patients have been treated with 10 g of pectin a day in the clinical study and no allergic symptoms have been observed. However, because pectin preparation procedures vary, these results should not be extrapolated to all pectin preparations that are commercially available.

## Data Availability

The datasets generated for this study are available on request to the corresponding author.

## Supplementary Materials

The following are available online at https://www.mdpi.com/article/10.3390/foods11010013/s1, Figure S1: Comparison of solubility of AP and CP in H_2_O, Fruktozym^®^-solution or Frutase^®^-solution (a). Impact of Fruktozym^®^ or treatment at 40 °C for 4 h on detectability and reactivity of nPru p 3 was examined. nPru p 3 (100 µg/mL), heat-treated nPru p 3, Fruktozym^®^-treated nPru p 3 as well as Fruktozym^®^ alone were analyzed by SDS-PAGE (left) and immunoblotting (right) using cross-reactive nsLTP rabbit antiserum (b). Figure S2: Spontaneous β-Hexosaminidase release from huRBL cells was examined by incubation with Fruktozym^®^, 5% AP + Fruktozym^®^ or 5% CP + Fruktozym^®^ (1:30 in H_2_O [*v/v*]) dilution series (a). Comparison of nPru p 3-dependent IgE-mediated β-Hexosaminidase release with and without prior Fruktozym^®^-treatment. Pru p 3 was diluted in Fruktozym^®^ (1:30 in H_2_O (*v/v*) (b).

## Abbreviations

AP	apple-derived pectin
CP	citrus-derived pectin
DE	degree of esterification
FCS	fetal calf serum
GalA	galacturonic acid
HG	homogalacturonan
HMP	high methoxyl pectin
LMP	low methoxyl pectin
MW	molecular weight
nPru p 3	natural Pru p 3
nsLTP	non-specific lipid transfer protein
OAS	oral allergy syndrome
RG	rhamnogalacturonan
XGA	xylogalacturonan
