# Supplementary material for: Does the Food Ingredient Pectin Provide a Risk for Patients Allergic to Non-Specific Lipid-Transfer Proteins?
Source: Foods. 2021 Dec 21;11(1):13. doi: 10.3390/foods11010013 (PMC8750200; doi:10.3390/foods11010013)
Supplement: Supplementary file 1 [file foods-11-00013-s001.zip › Supplemental Material.pdf]

Supplemental Material

Does the Food Ingredient Pectin Provide a Risk for Patients Allergic to Non-Specific Lipid-Transfer Proteins?

Hanna Steigerwald[1], Frank Blanco-Perez[1], Melanie Albrecht[1], Caroline Bender[2], Andrea Wangorsch[1], Hans-Ulrich Endreß[3], Mirko Bunzel[2], Cristobalina Mayorga[4], Maria José Torres[4], Stephan Scheurer[1,], Stefan Vieths[1]*

[1]Paul-Ehrlich-Institut (PEI), Federal Institute for Vaccines and Biomedicines, Langen, Germany

[2]Karlsruhe Institute of Technology (KIT), Institute of Applied Biosciences, Karlsruhe, Germany

[3]Herbstreith & Fox KG, Neuenbürg, Germany

[4]Allergy Unit, IBIMA-Regional University Hospital of Malaga-UMA, Malaga, Spain

a

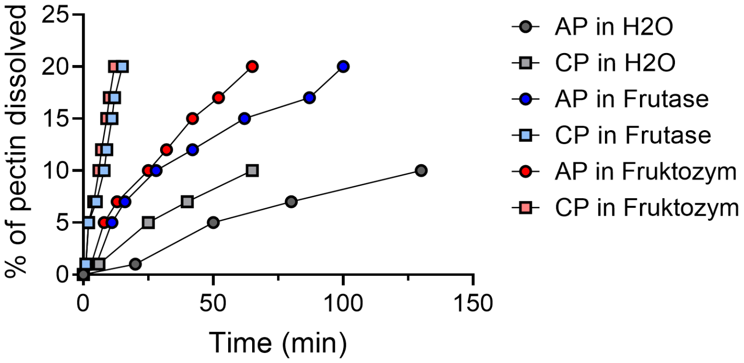

b

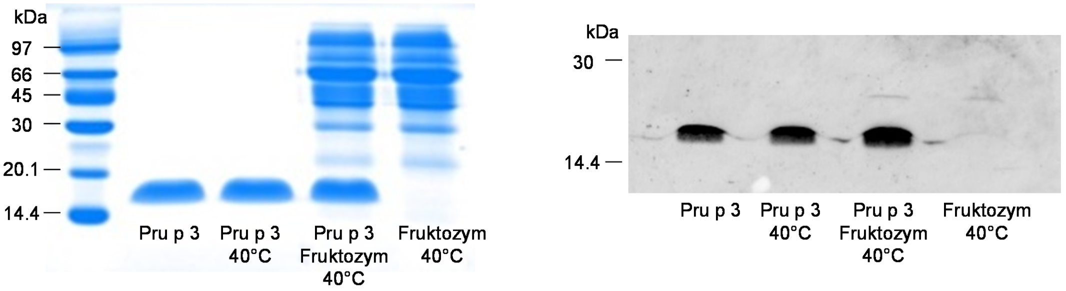

Figure S1: Comparison of solubility of AP and CP in H_2O, Fruktozym®-solution or Frutase®-solution (a). Impact of Fruktozym® or treatment at 40°C for 4 h on detectability and reactivity of nPru p 3 was examined. nPru p 3 (100 µg/ml), heat-treated nPru p 3, Fruktozym®-treated nPru p 3 as well as Fruktozym® alone were analyzed by SDS-PAGE (left) and immunoblotting (right) using cross-reactive nsLTP rabbit antiserum (b).

a

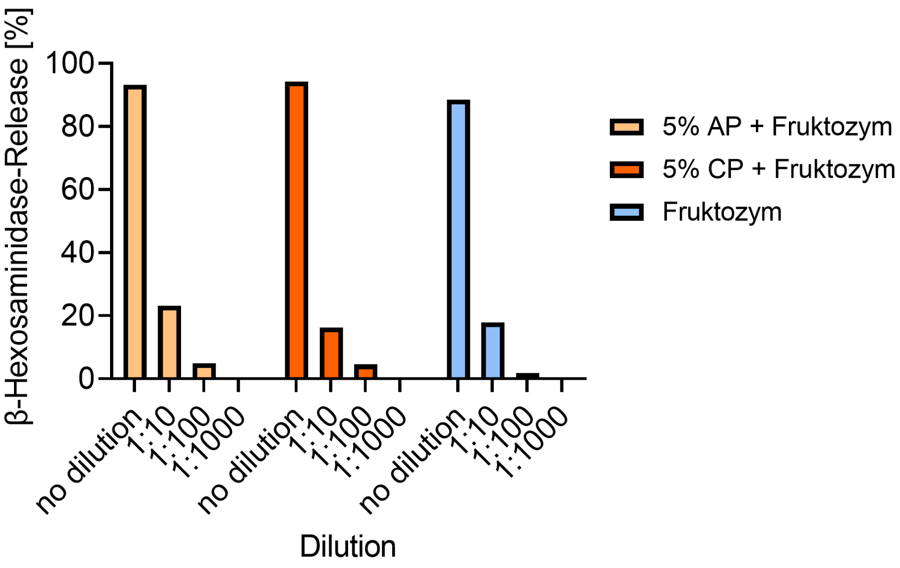

b

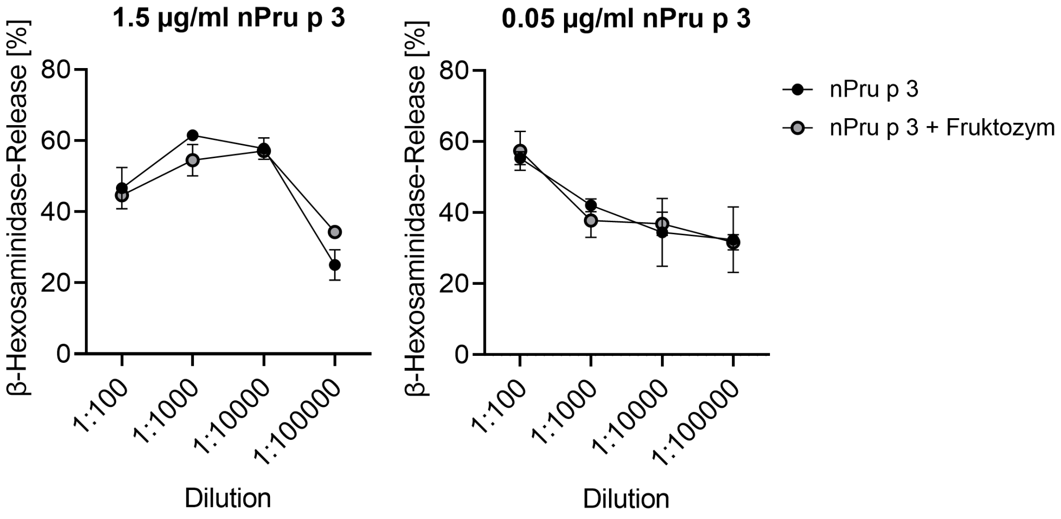

Figure S2: Spontaneous β-Hexosaminidase release from huRBL cells was examined by incubation with Fruktozym®, 5% AP + Fruktozym® or 5% CP + Fruktozym® (1:30 in H_2O [v/v]) dilution series (a). Comparison of nPru p 3-dependent IgE-mediated β-Hexosaminidase release with and without prior Fruktozym®-treatment. Pru p 3 was diluted in Fruktozym® (1:30 in H_2O [v/v]) (b).
